# Solitary Brain Metastasis: A Rare Initial Presentation of Prostate Carcinoma

**DOI:** 10.7759/cureus.4804

**Published:** 2019-06-02

**Authors:** Asim Hafiz, Muneeb Uddin Karim, Bilal M Qureshi, Adnan A Jabbar, Zubair Ahmad

**Affiliations:** 1 Radiation Oncology, Aga Khan University, Karachi, PAK; 2 Oncology, Aga Khan University, Karachi, PAK; 3 Pathology and Laboratory Medicine, Aga Khan University, Karachi, PAK

**Keywords:** brain metastasis, prostate carcinoma, radiation therapy, surgery, unusual presentation

## Abstract

Cerebral metastasis as an initial clinical presentation of prostate carcinoma is extremely rare. Usually, patients have widespread metastasis in the body before presenting with brain metastasis. In the absence of extensive metastasis, especially without bony metastasis, only brain metastasis is an unusual presentation of the disease. We report a case of a 59-years-old patient who presented with a lack of concentration and decreased vision. Magnetic resonance imaging (MRI) of the brain revealed a large right parietal-occipital space-occupying lesion. He underwent surgery and the pathological diagnosis of the tumor turned out to be metastatic prostate carcinoma. Further evaluation by a whole-body computed tomography (CT) scan revealed an enlarged prostate with no other metastatic deposit and a mildly raised level of prostate-specific antigen (PSA). It was possible for us to provide this patient with multi-modality treatment with the help of multidisciplinary tumor board meetings. Further studies addressing the biological as well as clinical characteristics of prostate carcinoma with this rare metastatic presentation will help us to define prognostic factors and therapeutic intervention and will help us to understand the basis of this unique presentation without bone metastasis.

## Introduction

Brain metastases are common in cancer patients; however, it presents as a late manifestation of the disease in patients with prolonged survival [1]. Prostate cancer commonly metastasizes to the pelvic lymph nodes and axial skeleton [2]. The occurrence of intracranial metastasis from prostate cancer was found to be <5% as reported in the literature comprising autopsy series [3]. Contemporary literature revealed <1% of prostate cancer patients with extensive disease developed brain metastasis [1]. But as an early clinical presentation of prostate adenocarcinoma without metastasis in the other parts of the body, this manifestation of the disease is extremely rare and is described in only a few case reports. Taking into consideration the rarity of this distinctive presentation, we are reporting a case to share our experience and provide further evidence of this unique entity.

## Case presentation

A 59-year-old male was brought to the emergency with a history of an acute headache secondary to a fall. He also complained of decreased vision, lack of concentration, and difficulty in walking for one month. Further history showed that he had lower urinary tract symptoms of decreased urinary frequency and poor urinary stream for six months, for which he was on follow up with a urologist outside our hospital. He also had essential hypertension and ischemic heart disease that was controlled on medication. He had no addiction. On examination, he was alert and vitally stable. Abdominal, heart, and lung examinations were unremarkable. Neurological examination revealed left-side homonymous hemianopia without ophthalmoplegia and tandem gait. Rectal examination revealed an enlarged prostate with no nodularity.

**Figure 1:**
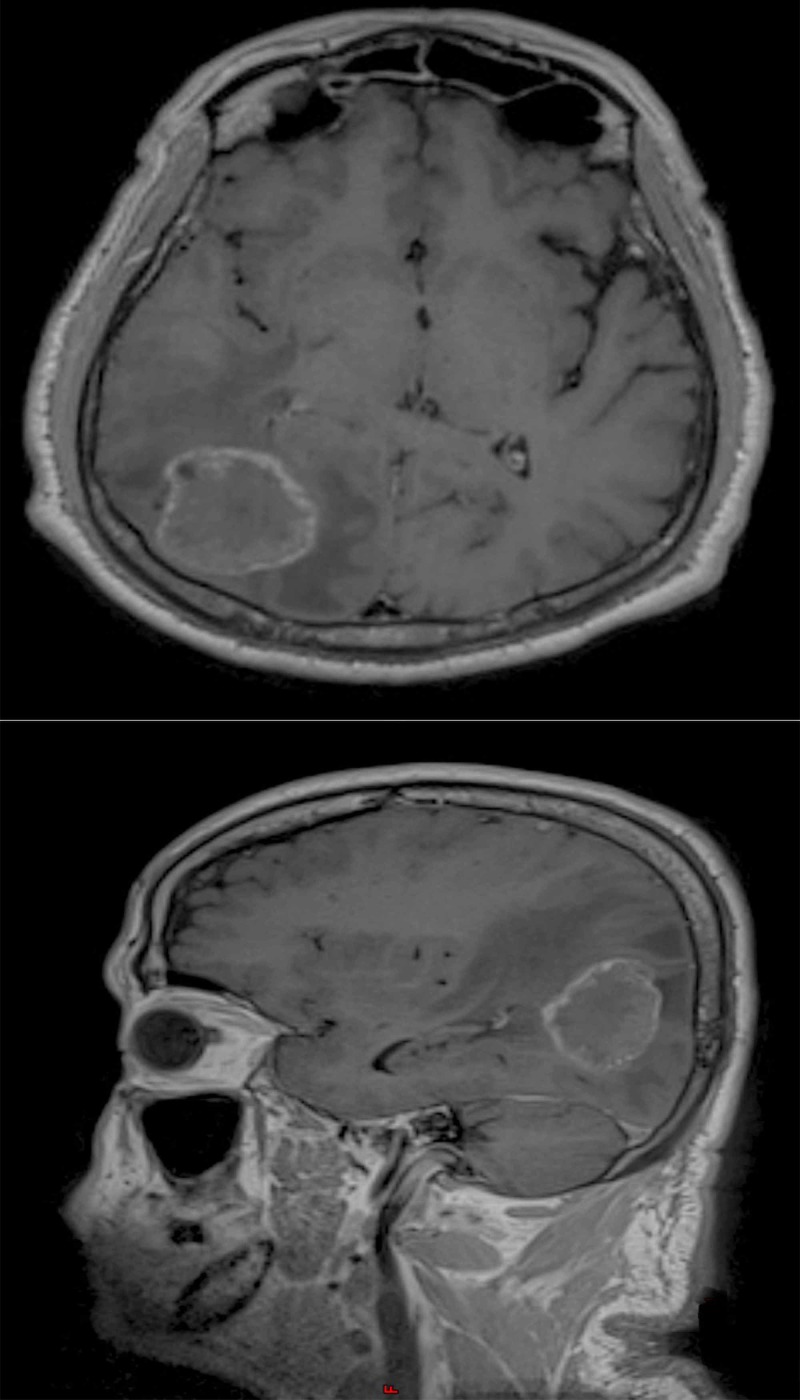
Preoperative MRI T1 sequence with contrast showing axial view (upper) and sagittal view (lower) of a space-occupying lesion in the occipital-parietal lobe

Magnetic resonance imaging (MRI) of the brain with contrast was performed, which revealed a focal well-defined hypo-intense lesion with poor contrast enhancement and significant peri-lesional edema in the right posterior parietal-occipital region (Figure 1).

**Figure 2:**
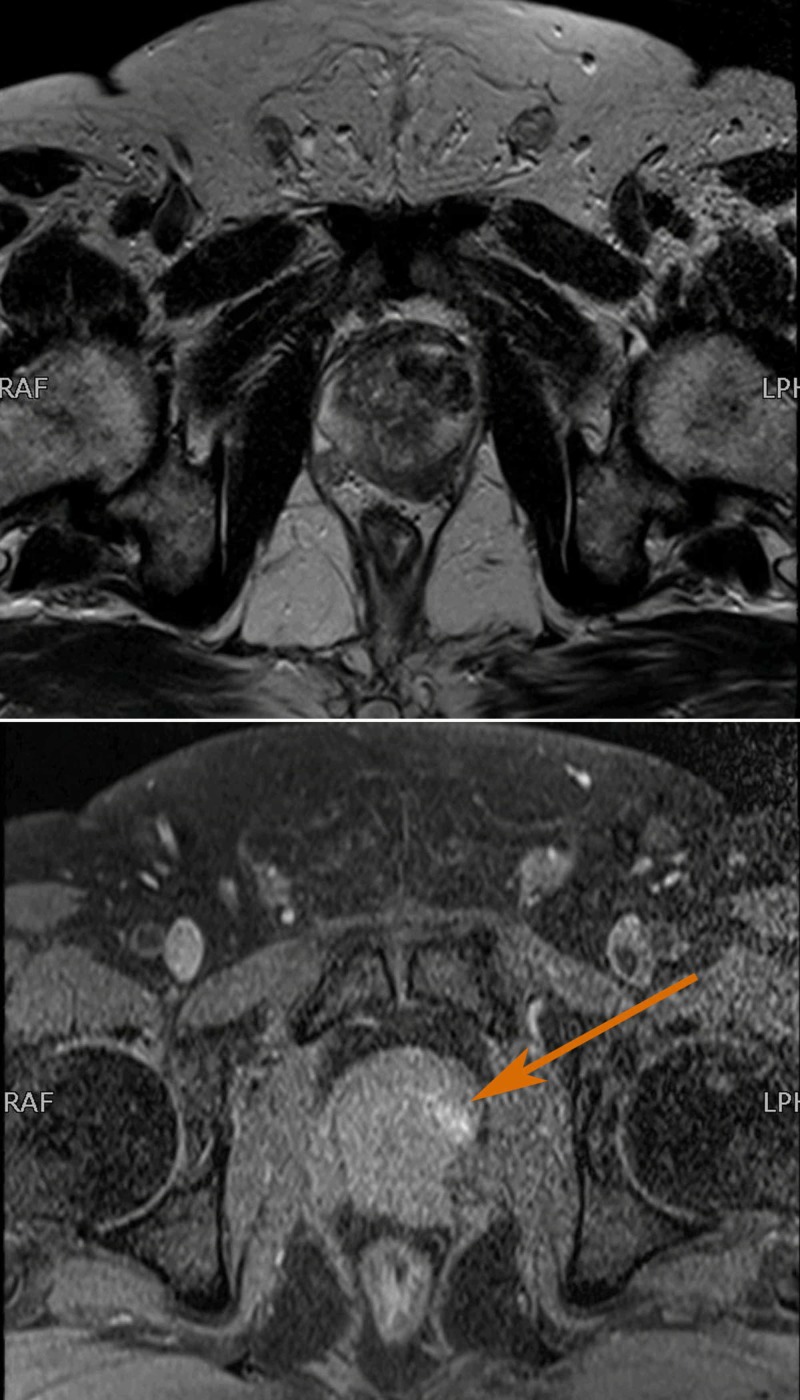
Pre-treatment MRI pelvis T1 with contrast (lower) and T2 (upper) axial images MIR showing abnormal signals identified in the left lobe of the prostate gland which predominantly involves the mid and basal zone and is crossing the midline. Posteriorly, on the left side, there is a focal breech in capsule and enhancement with central non-enhancing area suggestive of necrosis/haemorrhage.

Considering his history of urinary symptoms, an MRI of the pelvis was also done, which revealed an enlarged prostate gland showing abnormal heterogeneous T2 signals in the left lobe with a focal breech in the capsule and peri-prostatic lymph nodes. There was no definite involvement of the seminal vesicles (Figure 2).

The prostate-specific antigen (PSA) level turned out to be 8.5 ng/ml.

Metastatic workup was done under suspicion of prostate cancer, including bone scan and computed tomography (CT) chest abdomen and pelvis, which did not show any visible metastatic disease.

**Figure 3:**
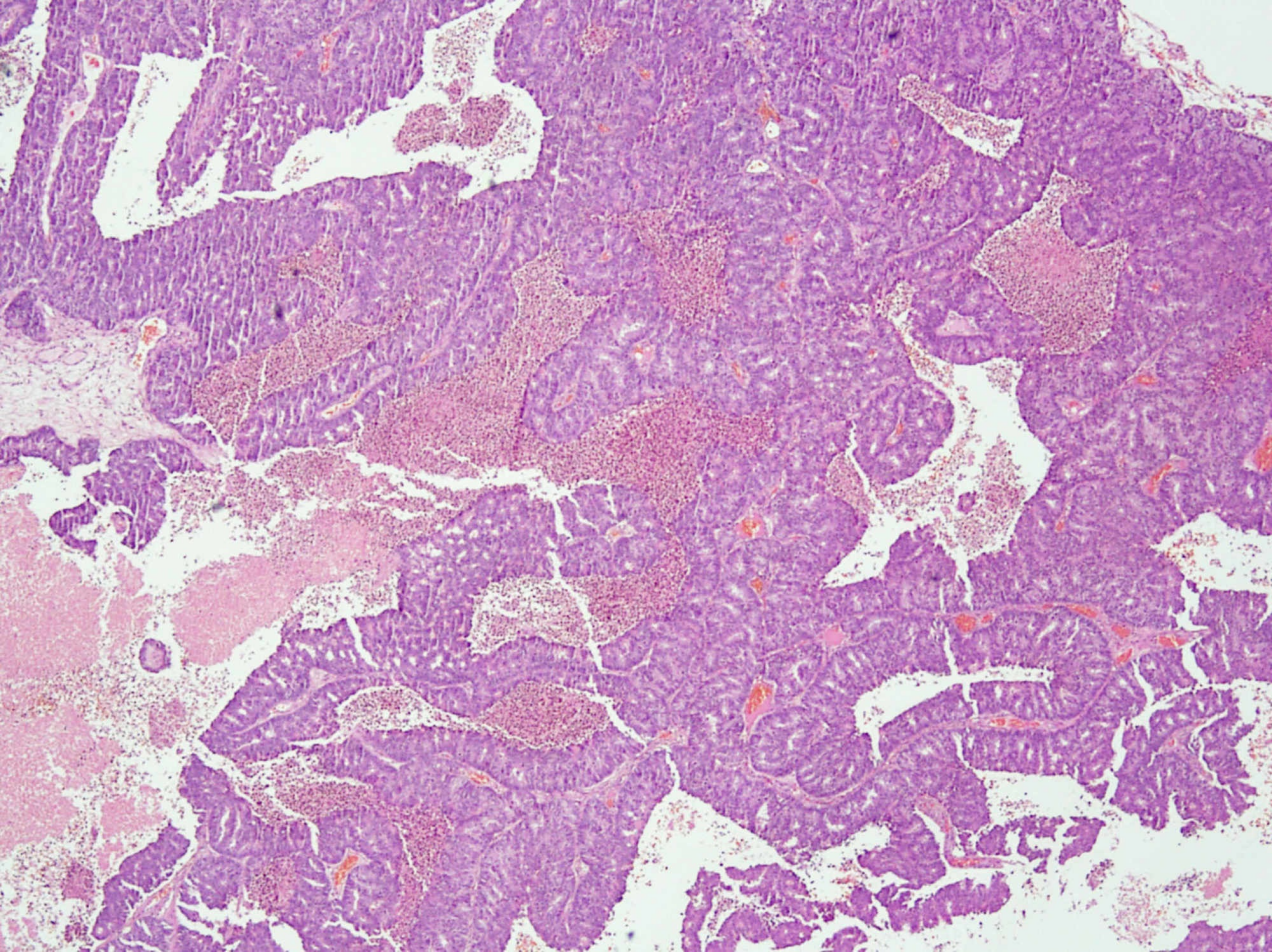
Histopathology: tumor infiltrating glial tissue H&E stain x10 H&E: haematoxylin and eosin stain

**Figure 4:**
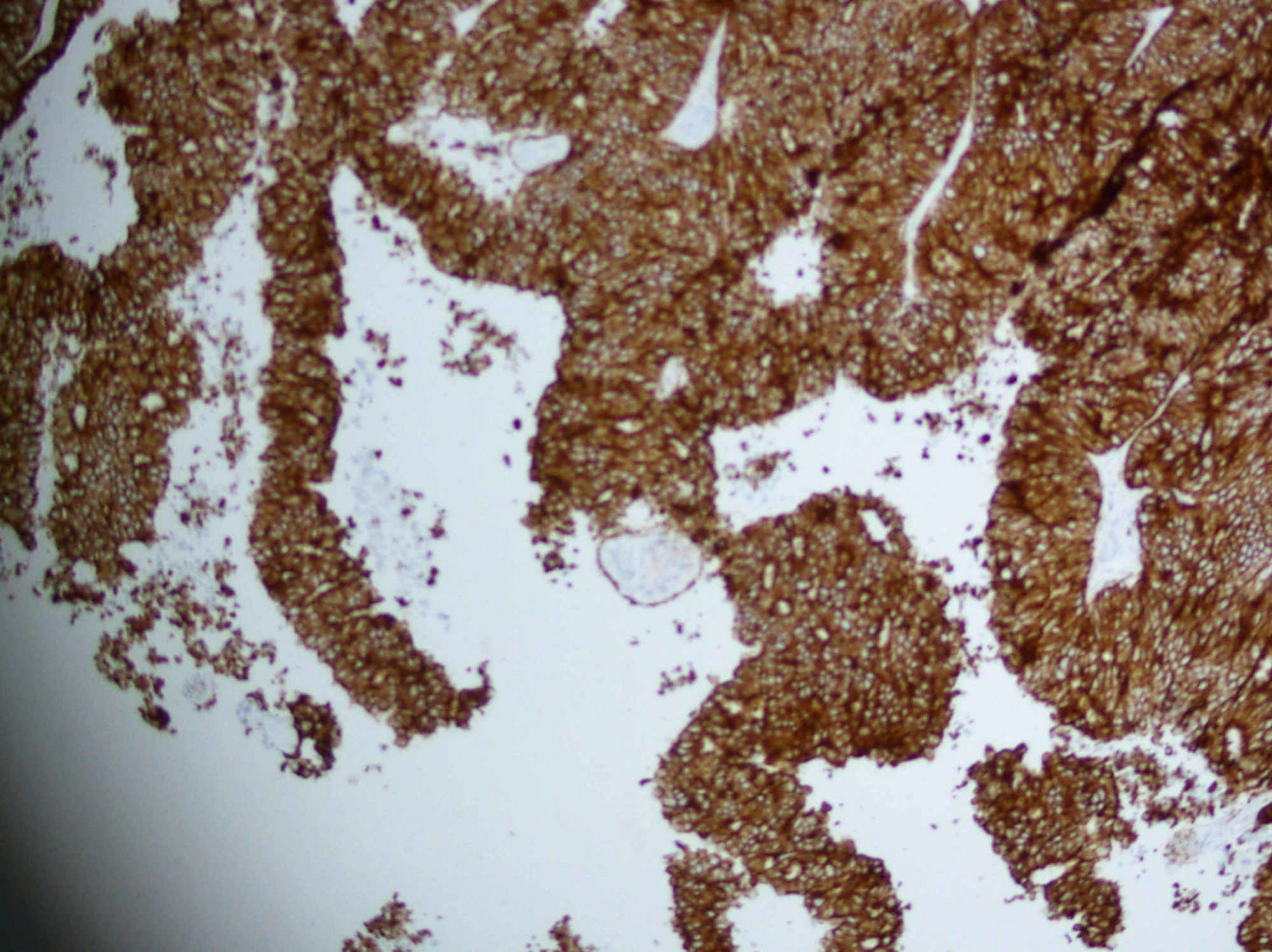
Histopathology: immunohistochemistry shows CK AE-1, AE-3 positivity original magnification x10 CK: Cytokeratin

**Figure 5:**
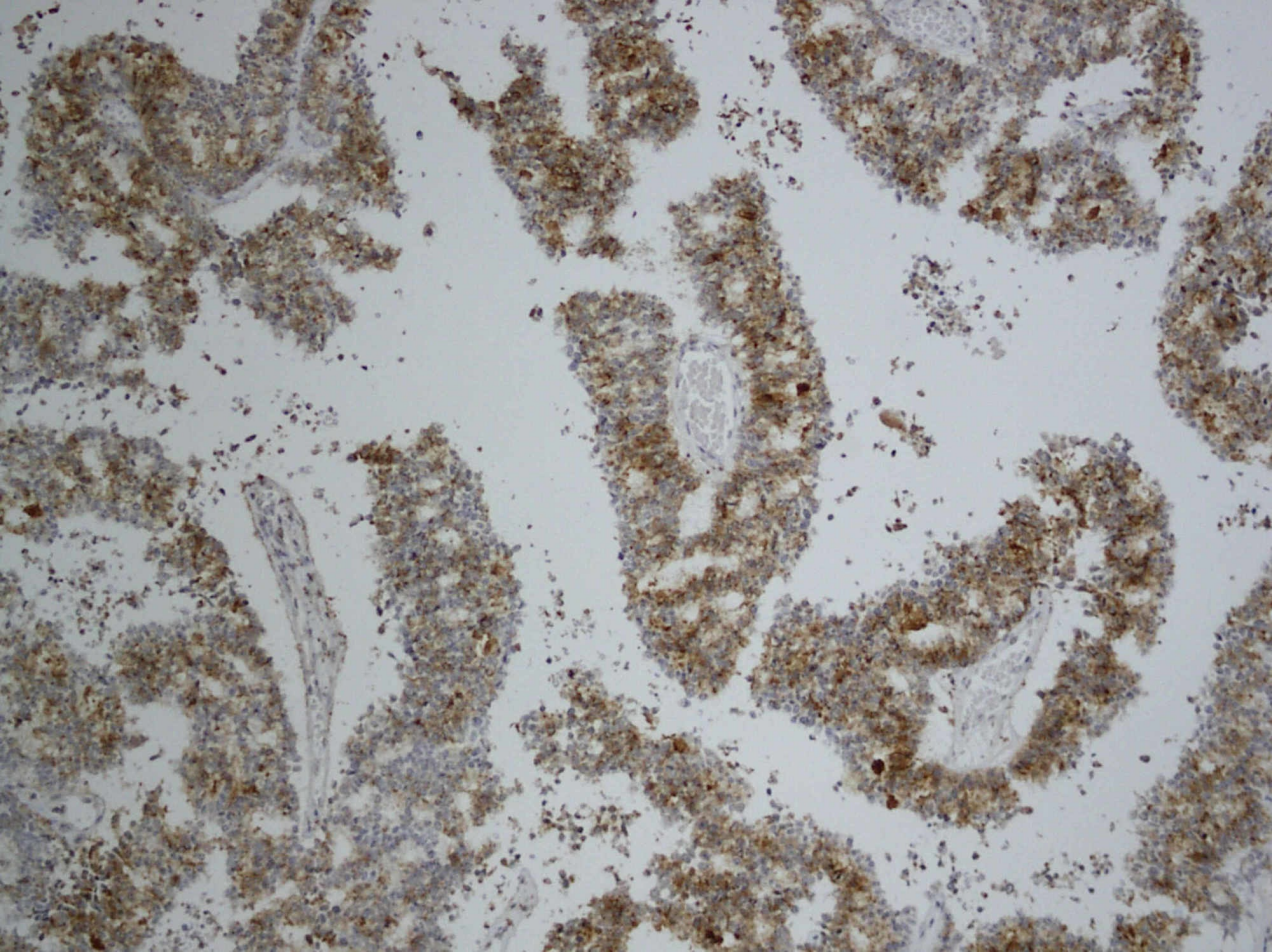
Histopathology: immunohistochemistry shows PSA positivity original magnification x10 PSA: Prostrate-specific antigen

Considering his acute symptoms, he was planned for surgical intervention first. He underwent neuro-navigation guided awake craniotomy and excision of the right parieto-occipital space-occupying lesion. Peroperatively, a highly vascular firm to soft lesion was found in the right parieto-occipital region. Postoperative MRI brain within 24 hours was suggestive of post-surgical changes with no definite evidence of residual disease. The patient recovered well postoperatively. Histopathological examination revealed a glial lesion composed of a glandular pattern with a cribri formation. These glands are lined by cuboidal to columnar cells having pleomorphic hyperchromatic nuclei and variably prominent nucleoli and moderate cytoplasm. The lumina of the gland also show areas of necrosis. Immuno-histochemical (IHC) staining shows cytokeratin (CK) AE1/AE3 positive, CK-7/20 negative, PSA positive, and synaptophysin and chromogranin negative. Considering the haematoxylin and eosin stain (H&E) and immunohistochemistry (IHC) finding, it was reported as metastatic adenocarcinoma with primary likely of prostate origin (Figures 3-5).

The patient subsequently underwent a trans-rectal ultrasound (TRUS) guided 12 core prostate biopsy that was consistent with prostate adenocarcinoma with Gleason’s score 4+4, sum 8/10 (prognostic grade group 4), involving all cores. His case was discussed in a multidisciplinary tumor board meeting and recommendation of radiation therapy and hormonal treatment was made. He received a radiation dose of 3000 cGy in 10 fractions to the whole brain. The patient was counseled on medical versus surgical options of hormonal treatment and the patient decided on the surgical option. He subsequently underwent bilateral subcapsular orchiectomy. 

**Figure 6:**
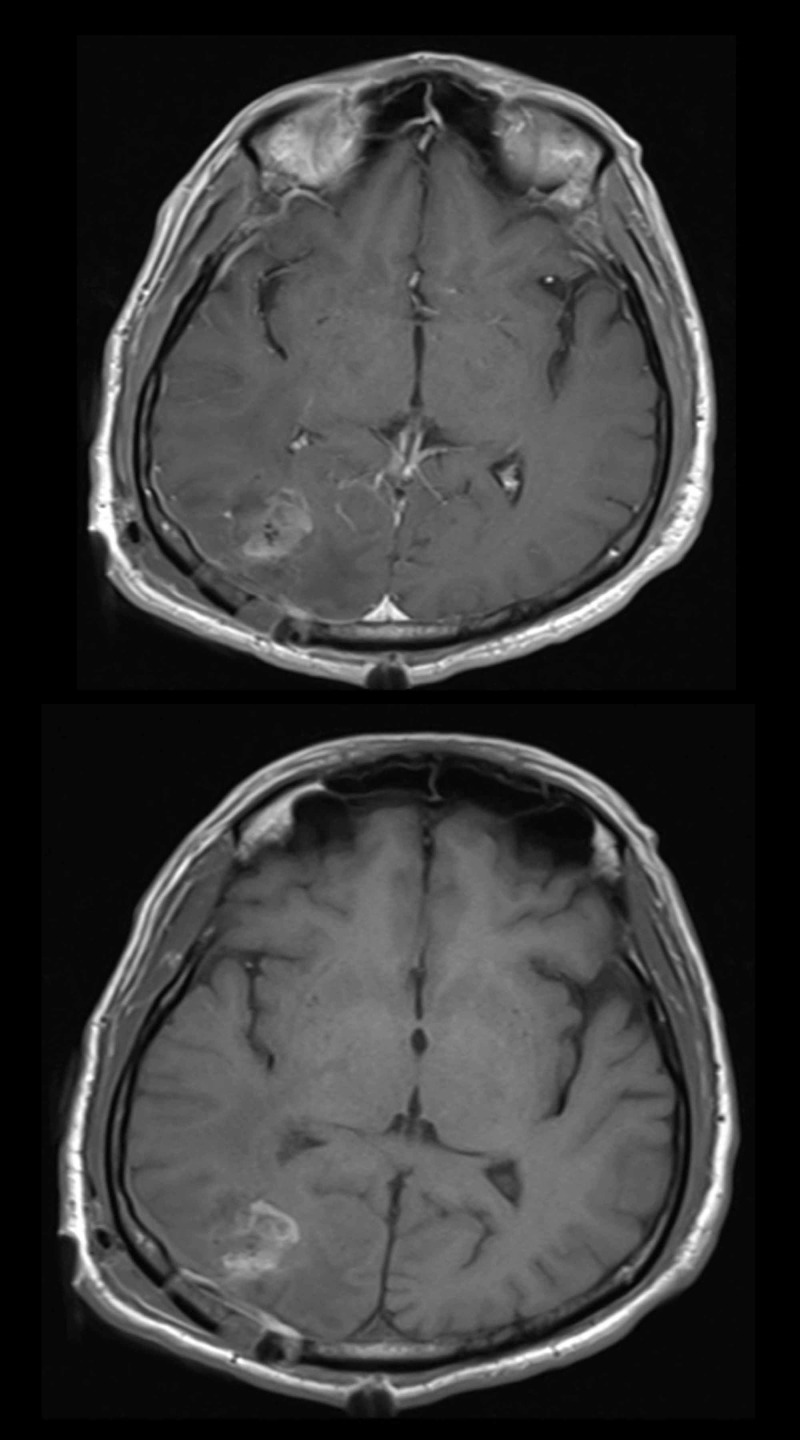
Follow up MRI T1 sequence with contrast (upper) and T1 sequence without contrast (lower) at 12 months showing no evidence of disease

Currently, the patient is on regular follow up and his recent PSA level is 3.31 ng/ml after one year of treatment and testosterone is at castration level (5.38 ng/dl). His MRI brain revealed no evidence of local recurrence (Figure 6). Bone scan is also negative for metastasis.

## Discussion

Brain metastasis from prostate carcinoma is infrequent and when present, it is often a late event of the disease and most patients have widespread metastasis elsewhere. Bone (axial skeleton) and lymph nodes (pelvic, retroperitoneal, and para-aortic) are the most frequent sites of spread. The lung is the most common visceral site otherwise noted at autopsy, but only 15%-20% of patients have extraosseous (soft tissue) metastases at the time of death [1,4]. A study by MD Anderson Cancer Centre examined the preferential metastatic sites in 316 patients with prostate cancer and found bone (89.5%) and abdominal lymph nodes (31.3%) to be the common sites involved; visceral metastases were less frequent, with lung (5.3%) and liver (4.1%) being the most common sites. An earlier study from the same institute reported that brain metastasis represented 1.6% of all metastases from prostate adenocarcinoma [1].

In contrast, a study based upon the Swedish registry reported that 9% of brain metastasis was found to have a prostate origin. However, this report was not specifically dedicated to assessing brain metastasis from prostate cancer but simply used the primary tumor recorded in the hospitals' clinical records without any further control, and so the findings should be viewed cautiously because of the possible demarcation of true vs. skull-base metastases, or even lepto-meningeal or dural metastases [5].

Cerebral metastases as an early clinical presentation of prostate adenocarcinoma are extremely rare [1,3,5-7]. Various hypothesis have been proposed about the mechanism of intracranial metastasis. The most common is the direct extension of skull metastases, but this leads to the development of subdural lesions. The second mechanism is the hematogenic spread, which can lead to subdural and intraparenchymal lesions. The third assumption suggests spreading through the low pressure paravertebral venous plexus that can directly seed the brain parenchyma without involving bone and any visceral sites [1,8]. Though adenocarcinoma is the most common histology associated with prostate cancer, certain histological types like a small cell or cribriform variant have a greater tendency for brain metastasis [1].

There is no distinct best treatment option for the management of brain metastasis from prostate cancer in the contemporary published literature. The aim of treatment should be to preserve a satisfactorily good quality of life for the patient. The established therapeutic approach for the treatment of central nervous system (CNS) metastasis from prostate cancer may involve radiotherapy, surgery, and radiosurgery alone or in combination. Androgen deprivation therapy is the primary therapeutic approach for advanced prostate cancer. It can be accomplished with orchiectomy, luteinising hormone-releasing hormone (LHRH) antagonist, or a combination of LHRH-antagonists plus an anti-androgen (complete androgen blockade) [3,8-10]. We deliberate that the treatment of choice for patients with brain metastases of prostate cancer is a combination of local treatments (radiotherapy, surgery and radiosurgery) with hormonal ablation therapy. We believe that with this rare metastatic clinical presentation of prostate carcinoma, further studies addressing the biology as well as clinical characteristics will help us to define prognostic factors and therapeutic interventions. Considering all these facts, we suggest a mandatory discussion in multidisciplinary site-specific tumor board meetings for the optimum management of all cases before embarking on treatment, in the absence of ideal published literature [11].

## Conclusions

Cerebral metastases as an initial clinical presentation of prostate adenocarcinoma without metastasis in other parts of body are extremely rare. Because of this unusual presentation of disease, further studies may be difficult to carry out, but global collaboration in health research, especially in this setting of rare presentation where the patients are dispersed, can play an integral role in furthering our understanding of the biological as well as clinical characteristics of prostate adenocarcinoma on the basis of this unique presentation. Owing to the rarity of these cases, the importance of having a mandatory discussion in multidisciplinary site-specific tumor board meetings, and having its recommendation on board before embarking on treatment, is evident for the best possible management of all cases.
